# Profiling the aromatic amino acid metabolome in the human gut microbiota reveals *Clostridioides difficile*-specific *N*-acyl amino acids

**DOI:** 10.1128/msystems.00779-26

**Published:** 2026-08-10

**Authors:** David Chun-Cheng Hsieh, Linhai Jiang, Mengzhao Xue, Niv Antonovsky, Sean F. Brady

**Affiliations:** 1 Laboratory of Genetically Encoded Small Molecules, The Rockefeller University 5929, New York, New York, USA; 2 Tri-Institutional PhD Program in Chemical Biology, The Rockefeller University 5929, New York, New York, USA; San Diego State University, San Diego, California, USA

**Keywords:** metabolism, mass spectrometry, *Clostridioides difficile*, gut microbiome, metabolomics, isotope tracing

## Abstract

Owing to its compositional and chemical complexity, much of the gut microbiota metabolome remains poorly characterized. Aromatic amino acids (AAAs) have a history of being privileged substrates for the biosynthesis of diverse bioactive metabolites and thus represent a potentially rich source of bioactive molecules within the microbiota metabolome. In this study, we leveraged ^13^C- and ^2^H-labeled aromatic amino acids and untargeted liquid chromatography-mass spectrometry (LC-MS) to profile AAA-derived metabolites produced by 80 phylogenetically diverse human gut bacterial isolates. Collectively, we found 93 unique LC-MS features, majority of which, predominantly produced by *Clostridioides difficile*, were identified as *N*-acyl amino acids. *C. difficile* produced the highest levels of the AAA-derived phenylacetic acid and phenylpropionic acid, exceeding all *Bacteroidetes* and *Proteobacteria* strains in our panel. *C. difficile*’*s* uniquely diverse *N-*acyl amino acids have the potential to serve as biomarkers for *C. difficile* colonization and mediators of *C. difficile*-specific host interaction.

**IMPORTANCE:**

The bacterial metabolome is a key component of the microbiota’s effect on host physiology, but identifying small molecules that potentially drive this interaction has remained a challenge. This study uses high-throughput and quantitative mass spectrometry metabolomics to show that *Clostridioides difficile* uniquely converts amino acids into at least 28 *N*-acyl amino acids, a metabolite family historically linked to diverse bioactivities. Additionally, in *C. difficile* cultures, high levels of phenylacetic acid, the precursor of 6 *N*-acyl amino acids, are of interest because previous studies have mechanistically linked microbially produced phenylacetic acid to cardiovascular disease via β2-adrenergic receptor (β2AR) signaling. The identification of species-specific metabolites produced by commensal bacteria provides not only compounds that could serve as sensitive biomarkers of colonization but also helps support the formulation of mechanistic hypotheses regarding how individual species influence their host.

## INTRODUCTION

The metabolome produced by the gut microbiota has the potential to be a key mediator of host-microbe interactions. As important as these metabolites may be to host physiology, the gut metabolome remains underexplored. This is underscored by over 10 million unique genes identified in human gut metagenomic sequencing and hundreds of potentially uncharacterized molecules seen in the microbiota-colonized specific pathogen-free (SPF) mice that are absent in germ-free animals (1, 2). Due to the complexity of this unknown molecular space, it is challenging to simultaneously analyze this entire metabolome even *in vitro*. Numerous mammalian and bacterial signaling molecules are derived from aromatic amino acids (AAAs). In fact, AAAs are considered privileged biosynthetic substrates as they require only limited transformation to produce ligands that interact with diverse molecular targets (1, 3–9). A focused search of AAA-derived metabolites produced by commensal bacteria therefore has the potential to identify bioactive metabolites at a higher frequency compared to a random search of an entire metabolome.

In this study, we combined stable isotope tracing and liquid chromatography-mass spectrometry (LC-MS) to develop a high-throughput metabolomics pipeline to identify the AAA metabolome across 80 diverse bacterial strains isolated from the human gastrointestinal tract. This screen revealed 93 species-resolved AAA-derived metabolite features. The majority of these metabolites, primarily produced by *Clostridioides difficile*, were *N-*acyl amino acids. *C. difficile* uniquely removed the amino group from amino acids to produce short mid-chain fatty acids, phenylacetic acid (PAA), and phenylpropionic acid (PPA), which were then conjugated with various amino acids to yield 28 distinct *N-*acyl amino acids. This unique *N*-acyl amino acid production was partly explained by the observation that *C. difficile* produces the highest levels of PAA and PPA among the strains we examined. As AAA-derived metabolites have a rich history of diverse bioactivities, the *C. difficile*-specific *N*-acyl amino acids we identified are prime candidates to explore for mediating *C. difficile*-specific host interactions. Our identification of numerous previously unknown AAA-derived MS features also shows the ability of stable isotope tracing to guide the identification of new metabolites and, in turn, the generation of testable hypotheses about how the microbiota may interact with its human host.

## RESULTS

### The aromatic amino acid metabolome of the gut microbiota

Metabolic redundancy across related bacterial species suggests that many common biologically active metabolites produced by commensal bacteria can be found by screening a phylogenetically diverse subset of the microbiota (10). To profile the AAA metabolome, we first curated a library of 80 human-associated bacteria acquired from the Biodefense and Emerging Infections Research Resources Repository (BEI Resources). Our final collection spanned the major phyla of gut microbiota, including six *Actinobacteria*, 22 *Bacteroidetes*, 38 *Firmicutes*, four *Fusobacteria*, and 10 *Proteobacteria*. We specifically included a subset of species that were known to metabolize AAAs (e.g., *Bifidobacterium longum*, *Clostridium cadaveris*, *Enterococcus faecalis*, and *Ruminococcus gnavus*).

**Fig 1:**
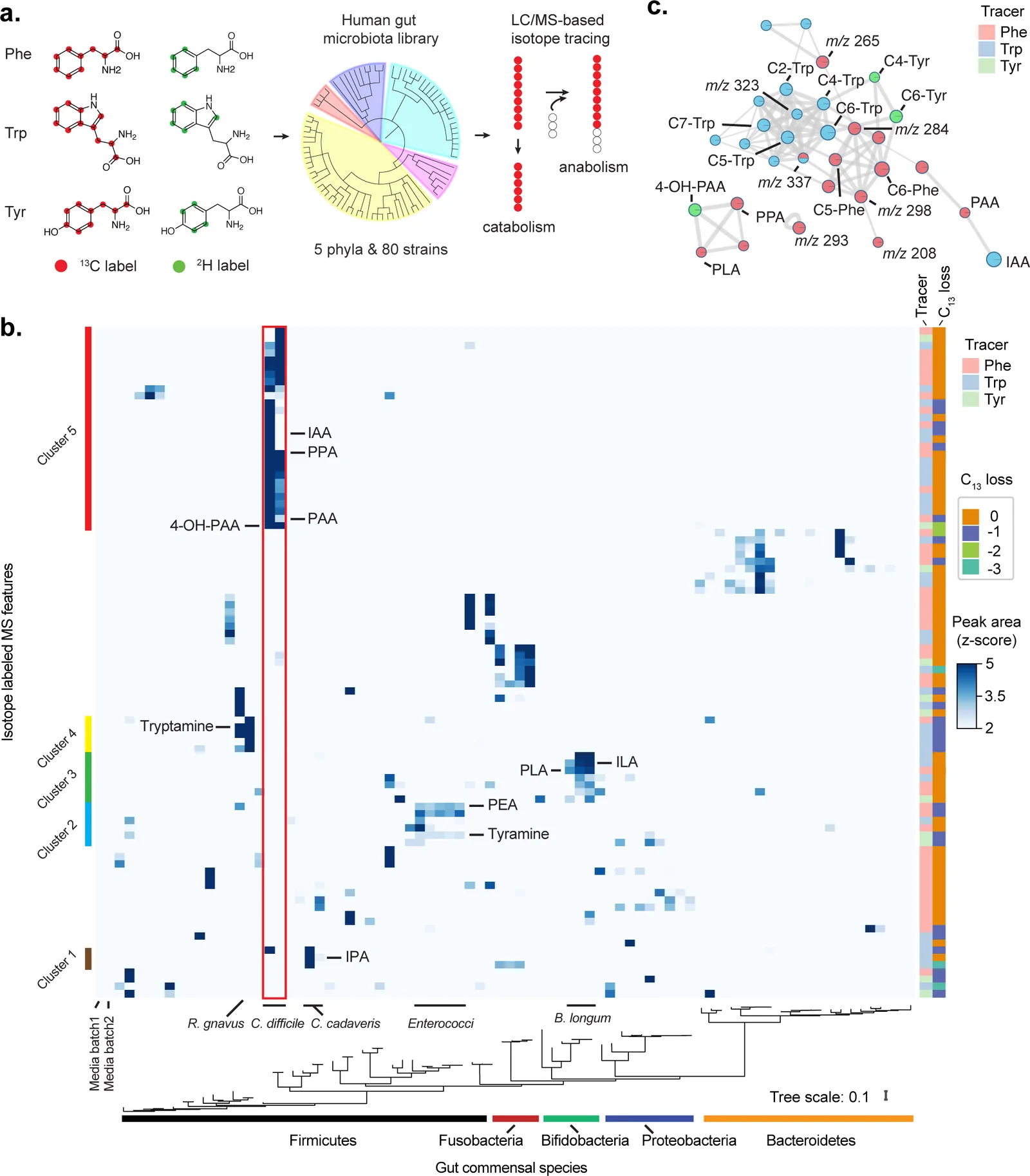
The aromatic amino acid metabolome of the human microbiota. (**a**) Schematic of our LC-MS based untargeted metabolomics protocol. Each bacterial species was cultured in medium containing the indicated stable isotopes of either phenylalanine, tryptophan, or tyrosine. (**b**) Heatmap of LC-MS features labeled by isotope tracers. The peak area for each feature was normalized across all bacterial species and shown as the average *z*-score. Strains are ordered based on a 16S rRNA phylogenetic tree. ^13^C loss indicates the difference between the detected number of ^13^C atoms and the number of ^13^C atoms in the fed isotope tracer. *n* = 3 per strain. Phe, phenylalanine; Trp, tryptophan; Tyr, tyrosine. (**c**) MS/MS-based molecular networking analysis of LC-MS features detected in the *C. difficile* culture. Nodes are connected based on their MS/MS similarity scores, and node size is proportional to the peak area of each feature.

To identify AAA-derived metabolites, each bacterium was individually grown (*n* = 3) in brain heart infusion (BHI) medium supplemented with pairs of ^13^C- or ^2^H-labeled aromatic amino acids (e.g., ^13^C- and ^2^H-labeled phenylalanine, tryptophan, or tyrosine) (Fig. 1a). The use of isotope labeling allows the differentiation of bacterially produced metabolites from those present in the medium. In addition to MS features that incorporated the full amino acid isotopes, we also looked for features that showed losses of ≤3 ^13^C and ≤2 ^2^H atoms to account for potential catabolism, while excluding features missing the entire amino acid side chain. Three-day-old culture broths were examined by LC-MS and untargeted metabolomic analyses using MetExtract II to identify ^13^C-labeled MS features (11).

All detected ^13^C-labeled features were cross-checked for a corresponding ^2^H-labeled mass in the same sample; features without a matching ^2^H-labeled mass were considered false positives and excluded. During MS ionization, a single metabolite can give rise to multiple signals including the protonated molecular ion [M + H]^+^, other adducts, and fragment ions. To better estimate the number of distinct AAA metabolites, features were removed if their masses appeared as fragments in the MS/MS (e.g., due to in-source fragmentation) or matched common adduct ions (e.g., multimers and Na^+^) of another feature at the same retention time (±0.1 min).

Our isotope labeling study identified 47 Phe-labeled, 35 Trp-labeled, and 11 Tyr-labeled features. The distribution of these features across all strains is displayed as a heatmap in Fig. 1b. Most of these features contained all atoms from the provided ^13^C and ^2^H isotopes, indicating incorporation of an intact amino acid into the labeled metabolites. MS/MS spectra of these features were searched against the MS/MS library from the Global Natural Products Social Molecular Networking (GNPS) database (12, 13). GNPS contains the largest publicly available database of natural product MS/MS spectra. A number of known microbiota metabolites were easily identified in our data, including tryptamine, tyramine, phenylethylamine (PEA), indoleacetic acid (IAA), indolepropionic acid (IPA), indolelactic acid (ILA), 4-hydroxyphenylacetic acid (4-OH PAA), phenylacetic acid (PAA), phenyllactic acid (PLA), and phenylpropionic acid (PPA).

All known metabolites contained the expected number of amino acid-derived isotope labels. For example, tryptamine, which was highly abundant in *R. gnavus* and *Lachnospiraceae bacterium* 2_1_58FAA cultures, was labeled by 10 ^13^C or 5 ^2^H atoms when the bacteria were cultured in medium supplemented with labeled tryptophan. We observed that known metabolites formed “hotspots” in the heatmap, indicating that most were produced by a small subset of microbiota species. This is consistent with previous findings that metabolic pathways are often phylum specific (4). Most features identified in our screen, however, did not match any previously reported metabolites in the GNPS MS/MS spectral libraries. Interestingly, expression patterns of several unannotated features were similar to known metabolites (Fig. 1b; clusters 1–5), suggesting that these features might share biosynthetic pathways with known metabolites.

### *C. difficile*-specific *N*-acyl amino acids are derived from AAAs

We prioritized structural elucidation of the AAA-associated metabolites from *C. difficile*, as it generated the greatest number of MS features among the strains we screened. MS/MS data from 31 features uniquely produced by *C. difficile* (those with a *z*-score greater than 1) were analyzed with GNPS molecular networking tools (12). Thirty MS features clustered into two networks, suggesting that their structures were similar (Fig. 1c). Nine of 26 features in the largest network matched MS/MS spectra of mid-chain saturated *N*-acyl amino acids. *N*-Acyl amino acids are common metabolites produced by mammals (14–18) and bacteria (19, 20) and are formed by the condensation between an amino acid and a fatty acid. Structurally diverse *N*-acyl amino acids have been found to affect varied biological processes, including thermogenesis, metabolism, neuronal function, and immunity (17, 18, 20–23).

**Fig 2:**
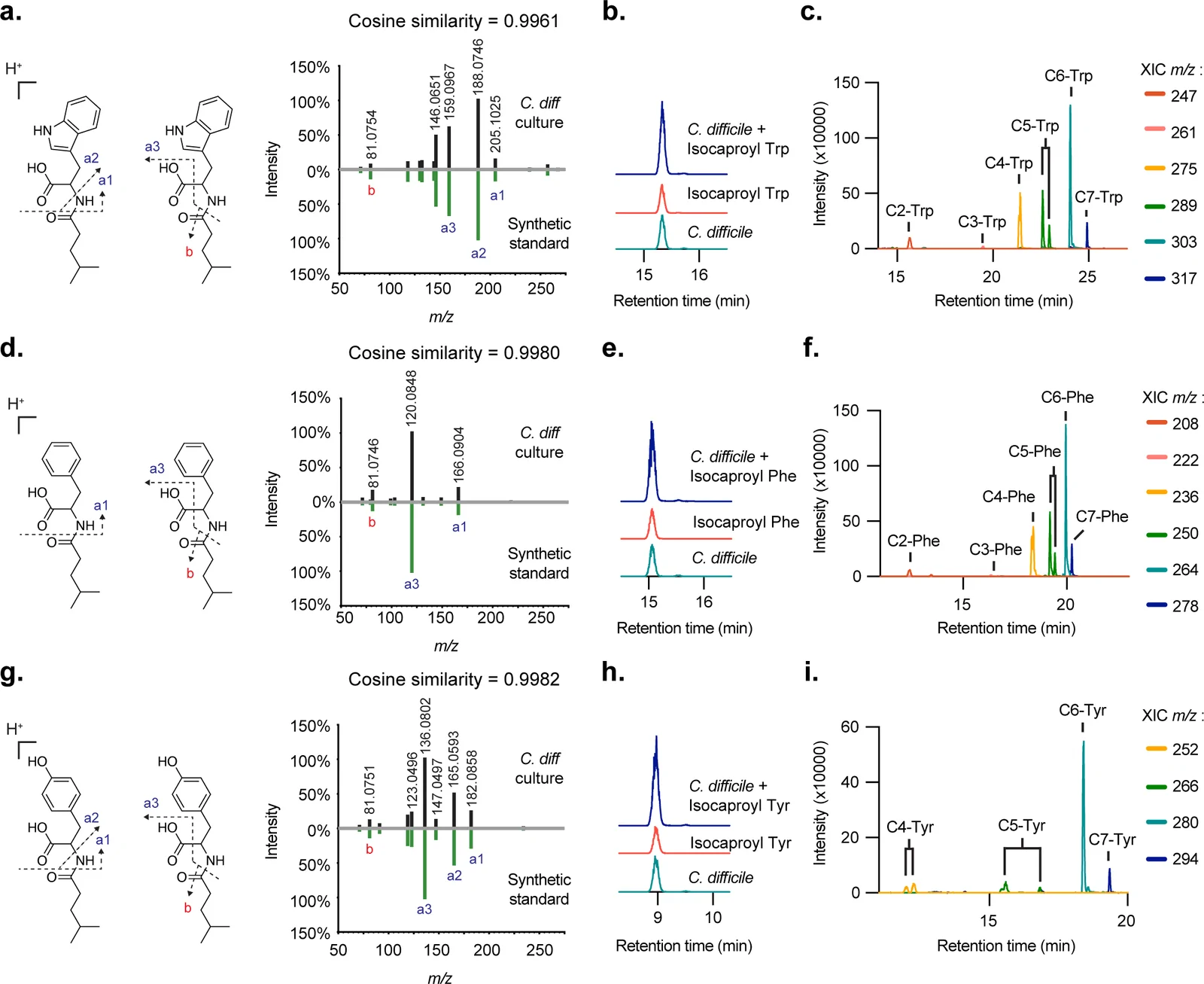
*C. difficile* produces *N*-acyl amino acids containing Stickland lipids. (**a, d, g**) Comparison of features from *C. difficile* HM-746 culture broth with MS/MS spectra of synthetic standards of (**a**) isocaproyl Trp, (**d**) isocaproyl Phe, or (**g**) isocaproyl Tyr. Structures of key fragment ions are shown: a1, intact amino acid fragment ions; a2, deaminated amino acid fragment ions; a3, decarboxylated amino acid fragment ions; b, isocaproyl fragment ions. (**b, e, h**) Comparison of extracted ion chromatograms (XIC) from *C. difficile* HM-746 culture with synthetic standards of (**b**) isocaproyl Trp, (**e**) isocaproyl Phe, and (**h**) isocaproyl Tyr. (**c, f, i**) Extracted ion chromatograms of (**c**) *N*-acyl Trp, (**f**) *N*-acyl Phe, and (**i**) *N*-acyl Tyr with the lipid tail length ranging from C2:0 to C7:0 in *C. difficile* HM-746 culture. Representative data are shown from three biological replicates.

MS/MS matching alone cannot distinguish lipid structural isomers, and therefore, we characterized the most abundant *N*-acyl amino acid feature produced by *C. difficile* in more detail. Based on MS/MS analysis alone, this feature matched C6-Trp (i.e., acyl group, C6:0; amino acid, tryptophan). We hypothesized that this acyl group was likely derived from either isocaproic acid or caproic acid, both of which are produced by *C. difficile* (24). Comparing the retention time and MS/MS spectra of synthetic caproyl Trp and isocaproyl Trp standards with those of the *C. difficile*-produced compound confirmed that the C6-Trp feature was isocaproyl Trp (iCTrp) (Fig. 2a and b; Fig. S1) (i.e., level 1 identification based on synthetic standard with matching retention time and MS/MS spectra) (25, 26). Similarly, we confirmed that features matching C6-Phe and C6-Tyr were isocaproyl Phe (iCPhe) and isocaproyl Tyr (iCTyr), respectively (Fig. 2d, e, g and h) (level 1 identification). The production of isocaproic acid analogs is not surprising, as *C. difficile* synthesizes isocaproic acid by reducing leucine via the Stickland reaction (24), and when *C. difficile* enters stationary phase, leucine is completely consumed, leading to millimolar concentrations of isocaproic acid in *C. difficile* cultures (27, 28). In addition to isocaproic acid-derived *N*-acyl amino acids, we observed lower levels of 13 other *N*-acyl amino acid features that correspond to lipid tails ranging from C2:0 to C7:0 (Fig. 2c, f and i; Fig. S2) (level 2 identification: putative compound based on MS/MS similarity to a standard). MS/MS analysis of these *N*-acyl amino acids produced the expected amino acid fragmentation patterns (Fig. S3 to S5). Consistent with the proposed structures, isotope labeling resulted in the expected increase in the *m*/*z* values of amino acid fragment ions, while acyl-group-derived fragment ions remained unchanged. This observation indicates that the acyl moieties were not derived from tryptophan, phenylalanine, or tyrosine. As *C. difficile* produces short-chain and mid-chain fatty acids (C5 to C7) from a variety of non-aromatic amino acids (24), we predict that these *N*-acyl amino acids were likely derived from the condensation of other Stickland-derived fatty acids with AAAs.

**Fig 3:**
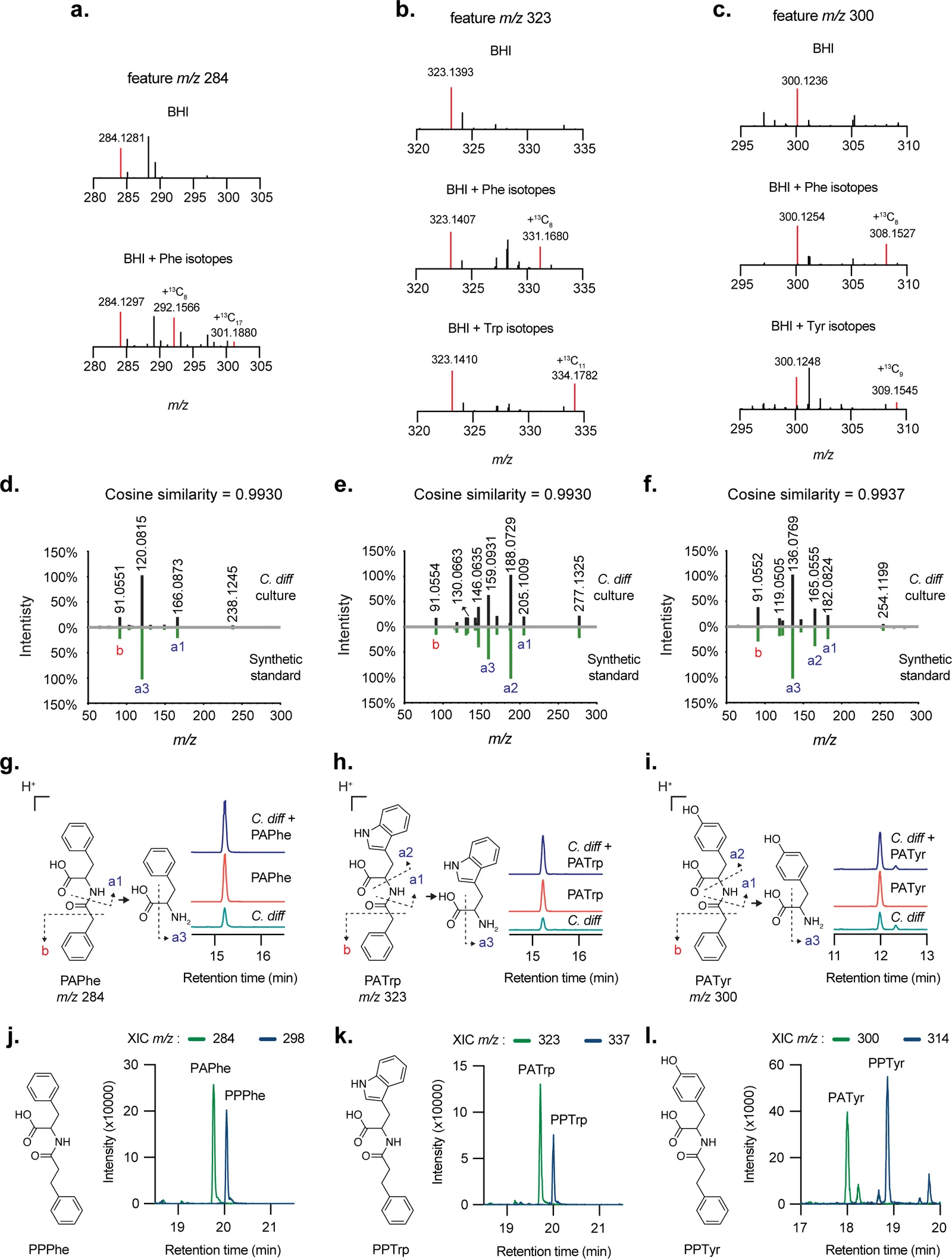
*C. difficile* produces *N*-acyl amino acids using PAA and PPA. (**a–c**) Isotope labeling of (**a**) PAPhe, (**b**) PATrp, or (**c**) PATyr features found in *C. difficile* HM-746 cultures. Masses of unlabeled and labeled metabolites are highlighted in red. (**d–f**) Comparison of MS/MS spectra from *C. difficile* HM-746 culture broth to synthetic standards of (**d**) PAPhe, (**e**) PATrp, or (**f**) PATyr. (**g–i**) Comparison of extracted ion chromatograms from *C. difficile* HM-746 culture broth with synthetic standards of (**g**) PAPhe, (**h**) PATrp, or (**i**) PATyr. Structures of key fragment ions in panels **g–i** are shown: a1, intact amino acid fragment ions; a2, deaminated amino acid fragment ions; a3, decarboxylated amino acid fragment ions; b, phenylacetyl fragment ions. (**j–l**) Extracted ion chromatograms of (**j**) PAPhe and PPPhe, (**k**) PATrp and PPTrp, and (**l**) PATyr and PPTyr in *C. difficile* HM-746 culture. Representative data are shown from three biological replicates.

After confirming that this large network consisted of *N*-acyl amino acids, we sought to characterize features within the network that were not identified in the GNPS database. Mass analysis of the most abundant feature (*m*/*z* 284.1281) indicated that the only plausible chemical formula within a 5-ppm error was C_17_H_18_NO_3_. All 17 carbons in this feature were labeled by ^13^C when *C. difficile* was grown with ^13^C_9_-phenylalanine (Fig. 3a). This suggested that the *m*/*z* 284.1281 feature was derived from two phenylalanine units, with the likely loss of one carbon through decarboxylation. MS/MS analysis of this feature revealed that it contained fragment ions corresponding to phenylalanine and an additional *m*/*z* 91.0535 fragment (Fig. 3d). MS/MS analysis of the ^13^C_17_-labeled *m*/*z* 284.1281 feature showed that the *m*/*z* of all fragment ions increased, suggesting that *m*/*z* 91.0535 was also derived from phenylalanine (Fig. S6). These data fit the characteristics of phenylacetyl phenylalanine (PAPhe), where the phenylacetic acid (PAA) originated from the labeled phenylalanine. The structure of *m*/*z* 284.1281 was confirmed by comparing the *C. difficile* metabolite with synthetic PAPhe (Fig. 3g) (level 1 identification). Similarly, using synthetic standards, we identified *m*/*z* 323.1393 as phenylacetyl tryptophan (PATrp) (Fig. 3b, e and h) and *m*/*z* 300.1236 as phenylacetyl tyrosine (PATyr) (Fig. 3c, f and i) (level 1 identification).

In addition to this set of PA-modified analogs, we found a related set of features with a +14 *m*/*z* shift corresponding to a CH_2_ addition: *m*/*z* 298.1432, 337.1558, and 314.1404 (Fig. 3j through l). In their MS/MS spectra, the parental +14 Da difference shifted the phenylacetyl fragment ion from *m*/*z* 91.0609 to *m*/*z* 105.0697 (Fig. S7), suggesting that these compounds contained a phenylpropionyl group in place of phenylacetyl. The number of isotope labels seen in each of these structures was consistent with the presence of both a phenylpropionyl and a corresponding amino acid substructure (Fig. S7a through c). Based on these data, we identified these features as phenylpropionyl phenylalanine (PPPhe), phenylpropionyl tryptophan (PPTrp), and phenylpropionyl tyrosine (PPTyr) (level 2 identification). Taken together, our data revealed that structures modified with diverse *N*-acyl substituents were the major aromatic amino acid metabolites produced by *C. difficile*.

### *C. difficile* produces diverse *N*-acyl amino acids

**Fig 4:**
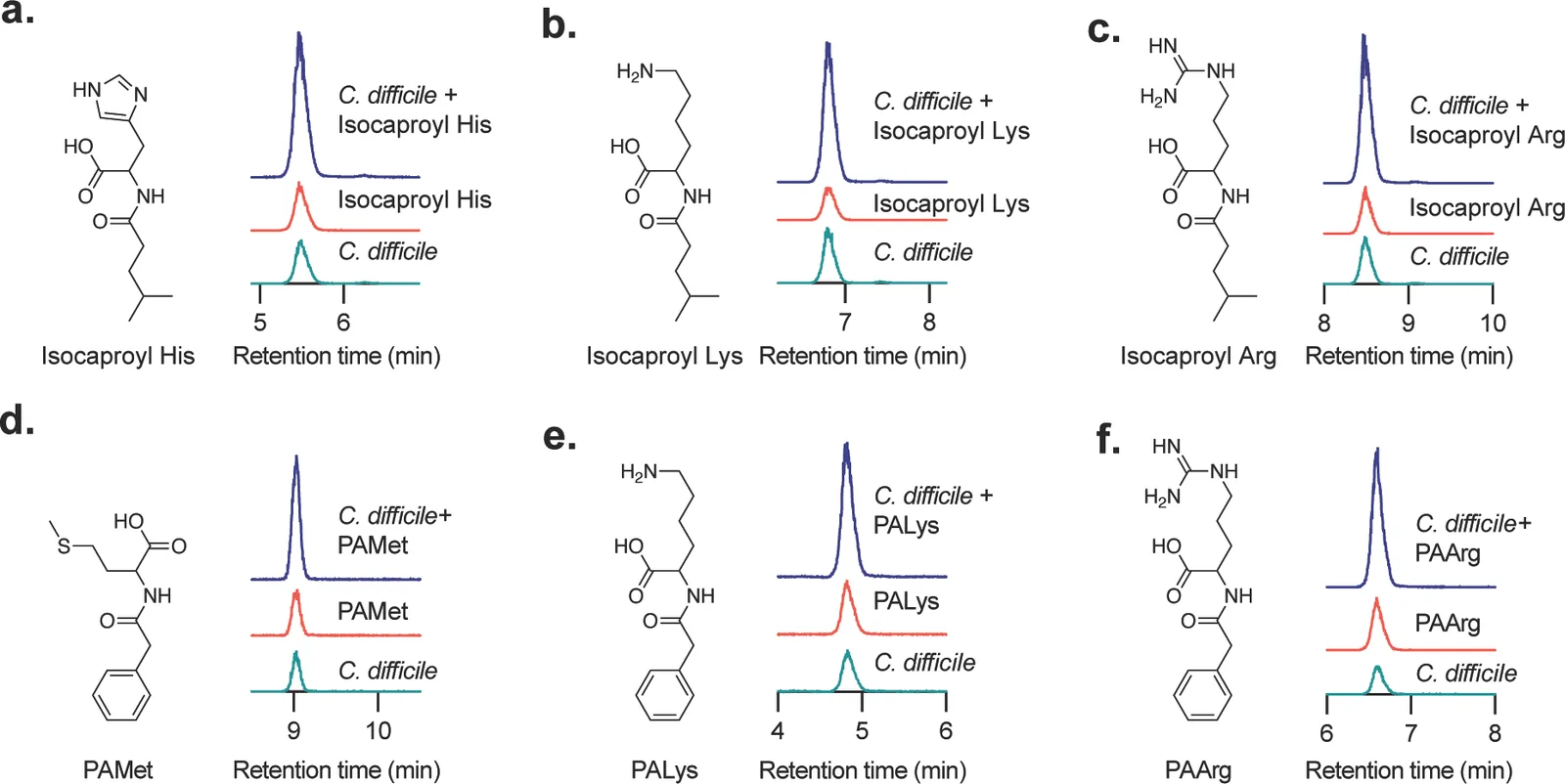
*C. difficile* produces *N*-acyl amino acids using diverse amino acids. (**a–f**) Comparison of extracted ion chromatograms from *C. difficile* HM-746 culture broth with synthetic standards of (**a**) isocaproyl His, (**b**) isocaproyl Lys, and (**c**) isocaproyl Arg, (**d**) PAMet, (**e**) PALys, or (**f**) PAArg. Representative data are shown from three biological replicates.

The discovery of *N*-acyl amino acids derived from all three aromatic amino acids motivated us to further screen *C. difficile* culture broth for other *N*-isocaproyl and *N*-phenylacetyl amino acids. To do this, we systematically searched for all masses corresponding to the condensation products of canonical amino acids with either isocaproic acid or phenylacetic acid. This search identified isocaproyl histidine (iCHis), isocaproyl lysine (iCLys), isocaproyl arginine (iCArg), phenylacetyl methionine (PAMet), phenylacetyl lysine (PALys), and phenylacetyl arginine (PAArg) (Fig. 4; Fig. S8) (level 1 identification).

### Across the gut microbiota, *C. difficile* produces the highest levels of phenylacetic acid and phenylpropionic acid

**Fig 5:**
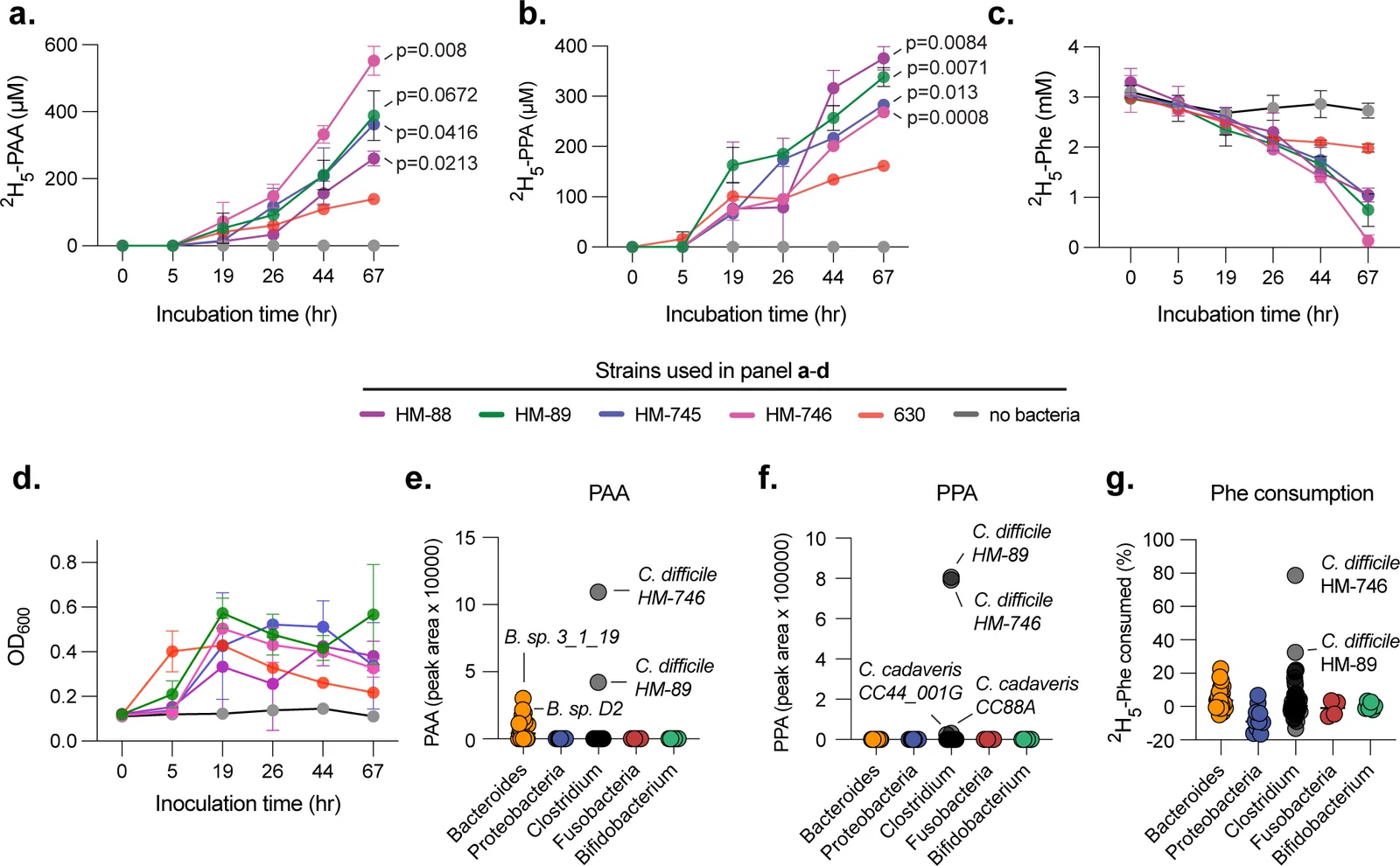
*C. difficile* is a major producer of PAA and PPA among the gut microbiota. (**a–d**) LC-MS quantification of (**a**) ^2^H_5_-PAA, (**b**) ^2^H_5_-PPA, and (**c**) ^2^H_5_-Phe levels in BHI medium supplemented with 3 mM ^2^H_5_-Phe. (**d**) Growth of *C. difficile* strains under these conditions. (**e–g**) LC-MS quantification of (**e**) PAA levels, (**f**) PPA levels, and (**g**) Phe consumption in the fermentation broths screened in Fig. 1. Data are shown as (**a–d**) the mean ± S.D. or (**e–g**) the mean from three biological replicates. *P*-values were calculated with mixed-effect analysis with Dunnett’s correction compared to strain 630. *P*-values for 67 h samples are labeled in panels **a and b**.

Next, we sought to examine the production of PAA and PPA by *C. difficile* in more detail as we speculated that the formation of PAA- and PPA-derived *N*-acyl amino acids was likely fueled by high levels of these two Phe catabolites. To test whether *C. difficile* can directly produce PAA and PPA, we performed LC-MS-based absolute quantification using 3 mM ^2^H_5_-phenylalanine as a tracer in five *C. difficile* strains, including the commonly studied strain 630. After 67 h, all five *C. difficile* strains produced more than 100 μM of isotope-labeled PAA and PPA (Fig. 5a and b). Notably, marked differences in PAA and PPA levels were found between strains, with strain 630 producing the least of both compounds. With high-level production of PAA and PPA, we observed corresponding consumption of phenylalanine by *C. difficile*. Nearly all phenylalanine was depleted by 67 h for strains HM-88, HM-89, HM-745, and HM-746 (Fig. 5c). *C. difficile* began to metabolize PAA and PPA only after reaching the maximum OD (Fig. 5d).

Interestingly, colonization of the gastrointestinal (GI) tract by PAA-producing microbes regulates the level of bioactive phenylacetyl glutamine (PAGln) (6). Increased levels of PAGln are functionally associated with a higher risk of several cardiovascular diseases, such as major adverse cardiovascular events (MACE), heart failure, and coronary atherosclerotic burden (6, 29). Our study of PAA and PPA in *C. difficile* cultures highlighted the strain-level variation in PAA and PPA-producing capacity. Given that the gut microbiota is highly heterogeneous, a large-scale cross-microbiota comparative study of producers would be interesting but is currently lacking. Such quantitative data would enable better modeling of microbiota metabolism of PAA, PPA, and their downstream metabolites, including PAGln and PPGln. We therefore re-analyzed our initial screening data to determine PAA, PPA, and ^2^H_5_-phenylalanine levels (Fig. 5e through g). Of note, *C. difficile* produced the highest levels of PAA even compared to previously recognized producers (e.g., *Bacteroides* and *Proteobacteria*) (Fig. 5e). In a human microbiome study, *Bacteroides*, *Proteobacteria*, and *Firmicutes* were identified as the major PAA and PPA producers because genes encoding either phenylpyruvate:ferredoxin oxidoreductase (PPFOR) or phenylpyruvate decarboxylase (PPDC) were enriched in these phyla (30). Furthermore, based on the link between PAA levels and cardiovascular disease, *ppfor* and *ppdc* are used as surrogate markers for atherosclerotic cardiovascular disease (ASCVD). Our results underscore that PAA production should not be inferred solely from the presence of PAA biosynthetic genes. Specifically, *C. difficile* HM-746 produced 3.6-fold higher PAA than *Bacteroides* sp. 3_1_19, the highest *Bacteroides* producer in our screen. While the biosynthesis of PAA has been observed in many species from *Proteobacteria* and *Bacteroides* (30), it has rarely been reported experimentally among *Firmicutes*, which aligns with our data that *C. difficile* was the only PAA producer among *Firmicutes* (Fig. 5e). For PPA, *C. difficile* produced 25-fold more than *C. cadaveris*, the only other PPA producer we found in our screen. Additionally, we found *C. difficile* was the only bacterial species that consumed more than 30% of labeled ^13^C-phenylalanine (Fig. 5g), further indicating that, within the microbiota, *C. difficile* may uniquely convert large quantities of Phe into PAA and PPA. The capability of PAA and PPA metabolism in *C. difficile* is reminiscent of *C. sporogenes*, another Firmicute species, whose production of similar aromatic amino acid derivative pairs (e.g., PAA, PPA, IAA, IPA, 4-OH PAA, and 4-OH PPA) has been mechanistically linked to intestinal permeability and host immunity (31). Taken together, our results show that *C. difficile* not only produces PAA and PPA but is also the highest producer among the gut microbiota species we tested.

## DISCUSSION

**Fig 6:**
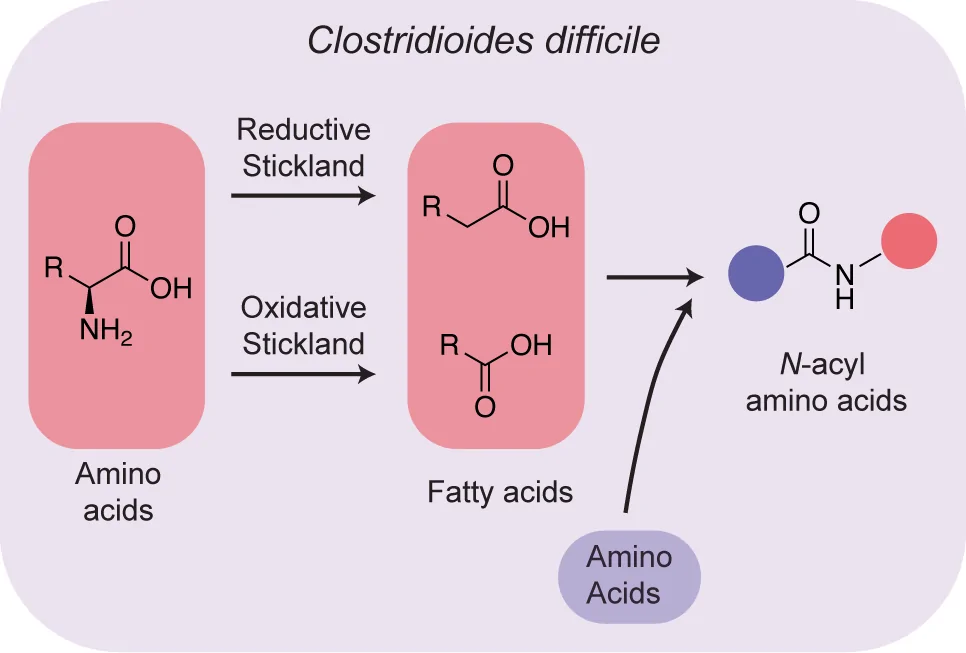
*N*-Acyl amino acid metabolism in *C. difficile*. *C. difficile* oxidizes and reduces amino acids via the Stickland reaction into various mid-chain fatty acids (e.g., isocaproic acid and phenylacetic acid). These newly formed fatty acids are conjugated with amino acids to form *C. difficile N*-acyl amino acids (e.g., isocaproyl Phe (iCPhe) and phenylacetyl Phe (PAPhe)).

Through our systematic untargeted metabolomic search of the AAA metabolome, we identified 93 AAA-derived metabolite features produced by the gut microbiota, demonstrating that aromatic amino acids are precursors to a rich diversity of commensal bacteria-produced metabolites. Among the 80 gut microbial species screened, *C. difficile* produced the greatest number of AAA-derived metabolites. This included a family of *N*-acyl amino acids that were exclusively produced by *C. difficile. C. difficile* uses mid-chain fatty acids derived from its Stickland metabolism to build *N*-acyl amino acids (Fig. 6). Our identification of these species-specific metabolites provides both potential biomarkers for *C. difficile* colonization and candidates for mediating *C. difficile*-specific effects on host physiology.

*C. difficile* displays a unique Stickland metabolism which utilizes amino acids to form large quantities of mid-chain fatty acids, including isocaproic acid and phenylacetic acid (24). Although C6:0 *N*-acyl conjugates have been detected in other human gut microbes (32), to the best of our knowledge, only one isocaproic acid-derived metabolite has been previously characterized in *C. difficile*: isocaproyl taurine, which is used as a biomarker for *C. difficile* infection (CDI) in the GI tract of patients with inflammatory bowel disease (IBD) (33). No *C. difficile* metabolite derived from phenylacetic acid has been reported. We identified six additional isocaproic acid as well as six phenylacetic acid-derived metabolites, suggesting that a focused search for metabolites that contain these Stickland products may be a rewarding avenue for identifying additional *C. difficile*-specific metabolites.

The potential bioactivities of the *C. difficile*-specific *N*-acyl amino acids remain to be determined; however, the analogy between some PA-amino acids and human-produced PAGln is intriguing. PAGln is a negative allosteric modulator (NAM) of the β2-adrenergic receptor (β2AR) (29). Functional (cAMP production and β-arrestin2 recruitment assays) and *in silico* docking analysis of WT and several mutants of β2AR revealed that PAGln binds to the same allosteric pocket (AS1) targeted by a synthetic NAM (29). While we did not detect PAGln in the *C. difficile* culture broth, it did produce several related structures in which Gln is replaced by Phe, Trp, Tyr, His, Arg, or Met. These metabolites share a high degree of structural similarity to PAGln. It remains to be seen whether any *C. difficile* PAGln analogs can engage β2AR or if they have other bioactivities. Interestingly, high levels of PAA and PPA produced by *C. difficile* could indirectly affect PAGln signaling. Previous mouse studies have found that colonization by other PAA-producing microbes (e.g., Proteobacteria and Bacteroides) can increase PAGln and PAGly levels in plasma and urine. As we found that *C. difficile* produced the highest levels of PAA and PPA among all bacteria screened, it is possible that *C. difficile* could similarly influence PAGln levels. This is an intriguing possibility as β2AR is highly expressed by cells at the primary *C. difficile* colonization site, including intestinal epithelium and gut mucosal immune cells (e.g.*,* B cells, macrophages, and group two innate lymphoid cells [ILC2s]) (34–36). These hypotheses remain speculative and require future investigation through direct studies using animal models or human samples. Nevertheless, our findings emphasize the power of stable isotope tracing to identify novel metabolites and, in turn, develop testable hypotheses about how the microbiota may affect its host.

## MATERIALS AND METHODS

### Chemicals

L-phenylalanine-^2^H_5_ (DLM-1258-1), L-phenylalanine-^13^C_9_ (CLM-2250), L-tryptophan-^2^H_5_ (DLM-1092), L-tryptophan-^13^C_11_ (CLM-4290), L-tyrosine-^2^H_4_ (DLM-451), and L-tyrosine-^13^C_9_ (CLM-2263) were purchased from Cambridge Isotope Laboratories. Isocaproic acid (277,827), phenylacetic acid (P16621), 2-chlorotrityl chloride resin, Fmoc-amino acids, piperidine, dichloromethane (DCM), dimethylformamide (DMF), 1-[bis(dimethylamino)methylene]-1H-1,2,3-triazolo[4,5-b]pyridinium 3-oxid hexafluorophosphate (HATU), triisopropylsilane (TIPS), trifluoroacetic acid (TFA), formic acid, acetonitrile, and diisopropylethylamine (DIPEA) were purchased from Sigma Aldrich. 3,3-Dimethylbutyryl Trp (Z239873492) and caproyl Trp (Z285192322) were purchased from Enamine.

### Bacterial culture

Bacterial strains are listed in Table S1. All bacteria were cultured anaerobically in a vinyl anaerobic chamber (Coy Laboratory Products) with a gas mixture of 5% CO_2_, 5% H_2_, and 90% N_2_ at 37°C. BHI medium consisted of 37 g/L brain heart infusion (Hardy Diagnostics), 5 g/L yeast extract, 200 mg/L MgSO4·7H_2_O, and 100 mg/L MnCl_2_·4H2O. After being autoclaved, 1 L of BHI medium was supplemented with 5 mL of 200 g/L maltose, 10 mL of 100 g/L cellobiose, 25 mL of 20 g/L-cysteine, and 5 mL of 1 g/L hemin. All media were deoxygenated by placing them in an anaerobic chamber for at least 24 h prior to use. Bacteria from a glycerol stock were plated on BHI agar, and a single colony was picked and cultured in BHI medium. For isotope-feeding experiments, 10 μL of the saturated 2-day culture was used to inoculate fresh 1 mL BHI medium supplemented with the indicated amino acid isotopes, followed by incubation for 3 days. L-tryptophan-^2^H_5_ and L-tryptophan-^13^C_11_ were spiked at a final concentration of 1 mM for tryptophan feeding. L-phenylalanine-^2^H_5_ and L-phenylalanine-^13^C_9_ were spiked at a final concentration of 3 mM for phenylalanine feeding. L-tyrosine-^2^H_4_ and L-tyrosine-^13^C_9_ were spiked at a final concentration of 0.5 mM for tyrosine feeding. These isotope concentrations were similar to endogenous amino acid concentrations in BHI medium. After incubation, the culture was centrifuged (10 min, 3,220 × *g*) to remove bacteria. Two hundred microliters of the resulting culture broth was added to 800 μL methanol containing 2 μM of oligomycin, followed by incubation at −80°C for 3 h and centrifugation (10 min, 3,220 × *g*) to precipitate proteins. From the resulting clarified supernatant, 800 μL was transferred to a 96-well plate, dried using a SpeedVac, and resuspended in 160 μL of a 1:1 mixture of methanol:water for LC-MS analysis. The OD_600_ of culture was measured using a plate reader (Tecan) at the indicated time.

### LC-MS for bacterial culture broth

The LC-MS analysis was performed using a Sciex X500R quadrupole time-of-flight MS coupled to an Exion LC. Turbo ESI source was set with the following parameters: curtain gas at 30 psi, ion source gas 1 at 50 psi, ion source gas 2 at 50 psi, temperature at 500°C, positive polarity, spray voltage at 5,500 V, CAD gas at 7, declustering potential at 80 V, collision energy at 5 V, TOF start mass at 100 Da, and TOF stop mass at 1,000 Da. MS/MS spectra were acquired with the same parameters, except that the collision energy was set to 30 V, the collision energy spread was set to 10 V, the TOF start mass was set to 20 Da, and TOF stop mass was set to 500 Da. Five microliters of spent culture was separated using a Waters Acquity UPLC BEH Shield RP18 1.7 μm, 2.1 × 150 mm column maintained at 40°C. The mobile phase consisted of buffer A (0.1% formic acid in water) and buffer B (0.1% formic acid in acetonitrile). The LC gradient for screening phenylalanine- and tyrosine-labeled features was as follows: 0–2 min, 2% B; 2–3 min, 10% B; 3–13 min, linear gradient from 10% to 18% B; 13–17 min, linear gradient from 18% to 50% B; 17–22 min, 95% B; and 22–30 min, 2% B. The flow rate was set at 0.25 mL/min. Tryptophan-labeled samples were analyzed using the following LC gradient: 0–2 min, 2% B; 2–3 min, 10% B; 3–15 min, linear gradient from 10% to 18% B; 15–23 min, linear gradient from 18% to 50% B; 23–26 min, 95% B; and 26–30 min, 2% B. We evaluated performance throughout the batch by monitoring carryover, retention time drift, and mass accuracy of the internal standard, oligomycin. Blank injections were analyzed every 24 samples to assess potential carryover (Fig. S9). For retention time matching experiments, the LC gradient for PATrp, PATyr, and PAPhe was as follows: 0–2 min, 5% B; 2–3 min, 5–10% B; 3–17 min, 10–55% B; 17–18 min, 55–95% B; 18–22 min, 95% B; and 22–30 min, 5% B. The LC gradient for isocaproyl Arg, isocaproyl His, isocaproyl Lys, PALys, and PAArg was as follows: 0–3 min, 5% B; 3–17 min, 5–20% B; 17–18 min, 20–95% B; 18–22 min, 95% B; and 22–30 min, 5% B. The LC gradient for PAMet, isocaproyl Phe, isocaproyl Tyr, isocaproyl Trp, and Fig. S1 was as follows: 0–3 min, 20% B; 3–17 min, 20–45% B; 17–18 min, 45–95% B; 18–22 min, 95% B; and 22–30 min, 20% B. PAMet was acquired using multiple reaction monitoring (MRM) due to its low signal, by monitoring the transition from *m*/*z* 268.1 to 91.0542. Each LC-MS file in wiff2 format was converted to mzXML format using MSConvert (ProteoWizard). If indicated, a synthetic standard was used to create a standard curve and run in the same batch to perform absolute quantification. Cosine similarity scores were calculated using Metabolomics Spectrum Resolver (37). The fragment tolerance was set to *m*/*z* 0.02.

### Isotope labeling analysis

#### MS feature identification

To identify ^13^C-labeled MS features (i.e., an *m*/*z* peak at specific retention time), the TracExtract module of MetExtract II (version 2.9.7) was used with the following parameters: atom count, 9; number of labeling atoms to search for, 6–9; native isotopolog, 12C; native isotopolog enrichment, 98.93%; labeled isotopolog, 13C; labeled isotopolog enrichment, 99%; amount native compound, 50; amount labeled compound, 50; ratio deviation relative to theoretical signal ratio, 50% to 170%; scan range, 1–28 min; intensity threshold, 10,000; charge, 1; mass deviation, ±5 ppm; isotopologs native, 2; isotopologs labeled, 2; maximum ratio deviation native, ±50%; maximum ratio deviation labeled, ±50%; MZ clustering window, ≤10 ppm; MZ clustering minimum spectra, ≥3 scans; XIC extraction width, ±10 ppm; XIC smoothing window, Gaussian and ±3 scans; chromatographic separation, 15–70 scans; EIC artificial shift, 0 scan; center error, ≤10 scans; and minimum corr, ≥85%. Tryptophan features were searched using the same parameters except atom count was 11, and the number of labeling atoms to search for was 8–11.

#### MS feature filtering

For each feature, expected ^2^H-labeled masses were calculated from the native mass (3 to 5 ^2^H for phenylalanine and tryptophan labeling; 2 to 4 ^2^H for tyrosine labeling). Peak areas of ^13^C-labeled, expected ^2^H-labeled, and native masses were extracted from the same LC-MS sample in which the feature was identified in MetExtract II using Mzmine 2 (38) (version 2.53) with the following parameters:

(i) Mass detection: MS level, 1; centroid mode; noise level, 3E3;
(ii) Targeted feature detection: intensity tolerance, 60%; noise level, 2E3; *m*/*z* tolerance, 20 ppm; retention time tolerance, 0.1 min (0.3 min for expected ^2^H-labeled masses);
(iii) Smoothing: filter width, 11.

All features were then aligned across samples using the following:

(i) Join aligner: *m*/*z* tolerance, 10 ppm; weight for *m*/*z*, 75; retention time tolerance, 0.3 min; weight for RT, 25;
(ii) Peak finder (multithreaded): intensity tolerance, 100%; *m*/*z* tolerance, 10 ppm; retention time tolerance, 0.2 min;
(iii) Peak filter: duration, 0.05 to 2.5 min; height, 2E3 to 1E8.

Features were kept based on the following criteria: (i) the difference in retention time between ^13^C-labeled and native masses was <0.1 min; (ii) the peak area ratio of (native mass/^13^C-labeled mass) was between 3 and 0.3; (iii) the retention time of ^2^H-labeled mass minus the retention time of native mass was between 0.1 and −0.3 min; and (iv) the peak area ratio of (^2^H-labeled mass/^13^C-labeled mass) was between 3 and 0.3. ^2^H-labeling number was determined by the largest peak area among all identified ^2^H-labeled masses. Finally, a feature was removed if any of the following criteria were met: (i) its ^2^H-labeled mass could not be found, (ii) its ^13^C-labeled mass was found in the BHI medium, and (iii) its native mass appeared as fragments in the MS/MS (e.g., due to in-source fragmentation) or matched a common adduct ion (e.g., multimers and Na^+^) of another feature at (±0.1 min) the same retention time. MS/MS spectra of native mass for each feature were extracted using the following parameters:

(i) Mass detection: MS level, 2; centroid; noise level, 1E1;
(ii) Targeted feature detection: intensity tolerance, 60%; noise leve,: 2E3; *m*/*z* tolerance, 20 ppm; retention time tolerance, 0.3 min.

### Molecular networking analysis

GNPS was used to analyze metabolite structural similarity among MS features based on MS/MS. The MS/MS spectra were filtered by removing all MS/MS fragment ions within ±17 Da of the precursor *m*/*z*. The precursor ion mass tolerance was set to 0.02 Da, and the MS/MS fragment ion tolerance was set to 0.02 Da. A network was then created in which edges were filtered to have a cosine score above 0.4 and more than four matched peaks. Furthermore, edges between two nodes were kept in the network if and only if each of the nodes appeared in each other’s respective top 10 most similar nodes. Finally, the maximum size of a molecular family was set to 100, and the lowest scoring edges were removed from molecular families until the molecular family size was below this threshold. The spectra in the network were then searched against GNPS' spectral libraries. The library spectra were filtered in the same manner as the input data. All matches kept between network spectra and library spectra were required to have a score above 0.7 and at least six matched peaks.

### 16S rRNA gene curation

Genome assemblies for each bacterial species were downloaded from NCBI using RefSeq assembly ID. The assembly data were processed with a Perl script using the BioPerl library to extract 16S rRNA sequences (39). For any bacterial species whose genome assembly is unavailable, 16S rRNA sequences were obtained by PCR using 27F (5′-AGAGTTTGATCMTGGCTCAG-3′) and 1492R (5′-GGTTACCTTGTTACGACTT-3′) primers, followed by Sanger sequencing using the 27F primer.

### Phylogenetic trees

Phylogenetic analysis was performed using NGPhylogeny (https://ngphylogeny.fr) with default settings and the following modules: multiple alignment, MAFFT; tree inference, PhyML; and tree rendering, Newick Display (40). The resulting tree was visualized using iTOL version 6 (https://itol.embl.de) (41).

### Synthesis of *N*-acyl amino acids

Fmoc-chemistry-based solid-phase peptide synthesis methods were used to synthesize all *N*-acyl amino acids at 23°C. Briefly, 120 mg of 2-chlorotrityl chloride resin (~0.1 mmol) was first swollen in dichloromethane (DCM) for 30 min, followed by the addition of 5 mL of a DCM solution containing 5 eq of the indicated amino acid and 10 eq of *N*,*N*-diisopropylethylamine (DIPEA). After 2 h of shaking, the resin was drained and washed three times with 5 mL DCM and 5 mL dimethylformamide (DMF), respectively. The Fmoc group was removed by shaking the resin with 10 mL of 40 vol% piperidine/DMF solution for 5 min, followed by extensive resin washing using DMF (6 × 5 mL). Next, a solution of the indicated lipid (isocaproic acid or phenylacetic acid) (5 eq), hexafluorophosphate azabenzotriazole tetramethyl uronium (HATU) (4.9 eq), and DIPEA (10 eq) in 3 mL DMF was added to the resin, and the mixture was shaken for 1 h. Afterward, the resin was washed with DMF (3 × 5 mL) and then DCM (3 x 5 mL). The resin was cleaved by shaking with a 2 mL cleavage cocktail composed of 95 vol% trifluoroacetic acid (TFA), 2.5 vol% water, and 2.5 vol% triisopropylsilane (TIPS) for 1 h. An oil-like crude product was then obtained by evaporating the cleavage cocktail using airflow and was subsequently dissolved in 3 mL of water and 3 mL of acetonitrile. The crude product was purified by reverse phase HPLC (Luna Prep C18, 5 µm, 21.2 × 150 mm, Phenomenex). The mobile phase consisted of solvent A (0.1 vol% formic acid in water) and solvent B (0.1 vol% formic acid in acetonitrile).

HRMS (ESI) analysis of isocaproyl Phe gave an [M + H]^+^ ion with a calculated *m*/*z* 264.1594 for C_15_H_22_NO_3_^+^ and a found *m*/*z* 264.1595.

HRMS (ESI) analysis of isocaproyl Trp gave an [M + H]^+^ ion with a calculated *m*/*z* 303.1703 for C_17_H_23_N_2_O_3_^+^ and a found *m*/*z* 303.1700.

HRMS (ESI) analysis of isocaproyl Tyr gave an [M + H]^+^ ion with a calculated *m*/*z* 280.1543 for C_15_H_22_NO_4_^+^ and a found *m*/*z* 280.1544.

HRMS (ESI) analysis of isocaproyl Lys gave an [M + H]^+^ ion with a calculated *m*/*z* 245.1860 for C_12_H_25_N_2_O_3_^+^and a found *m*/*z* 245.1857.

HRMS (ESI) analysis of isocaproyl Arg gave an [M + H]^+^ ion with a calculated *m*/*z* 273.1921 for C_12_H_25_N_4_O_3_^+^ and a found *m*/*z* 273.1918.

HRMS (ESI) analysis of isocaproyl His gave an [M + H]^+^ ion with a calculated *m*/*z* 254.1499 for C_12_H_20_N_3_O_3_^+^ and a found *m*/*z* 254.1502.

HRMS (ESI) analysis of PAPhe gave an [M + H]^+^ ion with a calculated *m*/*z* 284.1281 for C_17_H_18_NO_3_^+^ and a found m/z 284.1280.

HRMS (ESI) analysis of PATrp gave an [M + H]^+^ ion with a calculated *m*/*z* 323.1390 for C_19_H_19_N_2_O_3_^+^ and a found *m*/*z* 323.1382.

HRMS (ESI) analysis of PATyr gave an [M + H]^+^ ion with a calculated *m*/*z* 300.1230 for C_17_H_18_NO_4_^+^ and a found *m*/*z* 300.1229.

HRMS (ESI) analysis of PALys gave an [M + H]^+^ ion with a calculated *m*/*z* 265.1547 for C_14_H_21_N_2_O_3_^+^ and a found *m*/*z* 265.1550.

HRMS (ESI) analysis of PAArg gave an [M + H]^+^ ion with a calculated *m*/*z* 293.1608 for C_14_H_21_N_4_O_3_^+^ and a found *m*/*z* 293.1612.

HRMS (ESI) analysis of PAMet gave an [M + H]^+^ ion with a calculated *m*/*z* 268.1002 for C_13_H_18_NO_3_S^+^ and a found *m*/*z* 268.1000.

### Statistical analysis

Statistical analysis for each experiment can be found in the corresponding figure legends. All statistical analyses were performed using GraphPad Prism 10.

## Data Availability

Source data are provided with this paper. Key untargeted LC-QTOF metabolomics data are available at MetaboLights (https://www.ebi.ac.uk/metabolights/) under accession no. MTBLS13404. No custom code was used for this study. Requests for materials should be addressed to the corresponding author.

## SUPPLEMENTAL MATERIAL

Supplemental material msystems.00779-26-s0001.docx Figures S1 to S9; Table S1.

Table S2 msystems.00779-26-s0002.xlsx Source data.

ASM does not own the copyrights to Supplemental Material that may be linked to, or accessed through, an article. The authors have granted ASM a non-exclusive, world-wide license to publish the Supplemental Material files. Please contact the corresponding author directly for reuse.
