# Supplementary material for: Profiling the aromatic amino acid metabolome in the human gut microbiota reveals Clostridioides difficile-specific N-acyl amino acids
Source: mSystems. 2026 Aug 10;11(9):e00779-26. doi: 10.1128/msystems.00779-26 (PMC13595955; doi:10.1128/msystems.00779-26)
Supplement: Supplementary file 1 [file msystems.00779-26-s0001.docx]

**Supplementary information for:**

**Title: Profiling aromatic amino acid metabolome in the human gut microbiota reveals** ***Clostridioides difficile* specific *N*-acyl amino acids**

**Running title:** ***C. difficile* specific *N*-acyl amino acids from PAA**

**Authors:** David Chun-Cheng Hsieh^a,b^, Linhai Jiang^a^, Mengzhao Xue^a^, Niv Antonovsky^a^, Sean F. Brady^a#^

**Author affiliation**:

^a^Laboratory of Genetically Encoded Small Molecules, The Rockefeller University, New York, New York, USA

^b^Tri-Institutional PhD Program in Chemical Biology, The Rockefeller University, New York, New York, USA

**#Corresponding Author:** Sean F. Brady**,** [sbrady@rockefeller.edu](mailto:sbrady@rockefeller.edu)

Table of contents

1. Supplementary Table 1 Bacterial strains used in the study
2. Supplementary Table 2 Source data for Figure 1-5
3. Supplementary Figure 1 Extracted ion chromatograms (*m/z* 303.1703) of caproyl Trp, 3,3-dimethyl butyryl Trp, and *C. difficile* culture.
4. Supplementary Figure 2. Isotope labeling analyses of additional *N*-acyl amino acid features found in *C. difficile* culture shown in Figure 2.
5. Supplementary Figure 3. MS/MS analyses of unlabeled and isotope labeled *N*-acyl phenylalanine features found in *C. difficile* culture shown in Figure 2.
6. Supplementary Figure 4. MS/MS analyses of unlabeled and isotope labeled *N*-acyl tryptophan features found in *C. difficile* culture shown in Figure 2.
7. Supplementary Figure 5. MS/MS analyses of unlabeled and isotope labeled *N*-acyl tyrosine features found in *C. difficile* culture shown in Figure 2.
8. Supplementary Figure 6. Isotope labeled MS/MS analyses of *N*-phenylacetyl amino acid features found in C. difficile culture shown in Figure 3.
9. Supplementary Figure 7. MS/MS and isotope labeling analyses of *N*-phenylpropionyl amino acid features found in *C. difficile* culture shown in Figure 3.
10. Supplementary Figure 8. Comparison of MS/MS spectra of *N*-isocaproyl amino acids and *N*-phenylacetyl amino acids between *C. difficile* HM-746 culture broth to synthetic standards.
11. Supplementary Figure 9. Internal standards showed consistent LC-MS performance across the screen.

| **BEI HM#** | **NCBI:txid (as of 07162022)** | **Strain** | **phylum** |
| --- | --- | --- | --- |
| **633** | 411481 | Bifidobacterium adolescentis -- L2-32 | Actinobacteria |
| **847** | 1161904 | Bifidobacterium longum subsp. longum -- 35B | Actinobacteria |
| **845** | 1161743 | Bifidobacterium longum subsp. longum -- 44B | Actinobacteria |
| **30** | 216816 | Bifidobacterium sp. -- 12_1_47BFAA | Actinobacteria |
| **304** | 742722 | Collinsella sp. -- 4_8_47FAA | Actinobacteria |
| **1055** | 1073369 | Rothia mucilaginosa -- CC87LB | Actinobacteria |
| **728** | 997873 | Bacteroides caccae strain CL03T12C61 | Bacteroidetes |
| **726** | 997874 | Bacteroides cellulosilyticus -- CL02T12C19 | Bacteroidetes |
| **719** | 997876 | Bacteroides dorei -- CL02T12C06 | Bacteroidetes |
| **718** | 997877 | Bacteroides dorei -- CL03T12C01 | Bacteroidetes |
| **29** | 556260 | Bacteroides dorei strain 5_1_36/D4 | Bacteroidetes |
| **210** | 665953 | Bacteroides eggerthii -- 1_2_48FAA | Bacteroidetes |
| **20** | 457424 | Bacteroides fragilis -- 3_1_12 | Bacteroidetes |
| **709** | 997882 | Bacteroides fragilis -- CL07T00C01 | Bacteroidetes |
| **710** | 997883 | Bacteroides fragilis -- CL07T12C05 | Bacteroidetes |
| **714** | 997879 | Bacteroides fragilis strain CL03T12C07 | Bacteroidetes |
| **711** | 997880 | Bacteroides fragilis strain CL05T00C42 | Bacteroidetes |
| **222** | 665954 | Bacteroides ovatus -- 3_8_47FAA | Bacteroidetes |
| **22** | 457387 | Bacteroides sp. -- 1_1_30 | Bacteroidetes |
| **23** | 818 | Bacteroides sp. -- 1_1_6 | Bacteroidetes |
| **18** | 469588 | Bacteroides sp. -- 2_1_22 | Bacteroidetes |
| **19** | 469592 | Bacteroides sp. -- 3_1_19 | Bacteroidetes |
| **27** | 457395 | Bacteroides sp. -- 9_1_42FAA | Bacteroidetes |
| **28** | 556259 | Bacteroides sp. -- D2 | Bacteroidetes |
| **189** | 585543 | Bacteroides sp. -- D20 | Bacteroidetes |
| **720** | 997891 | Bacteroides vulgatus strain CL09T03C04 | Bacteroidetes |
| **731** | 999419 | Parabacteroides johnsonii -- CL02T12C29 | Bacteroidetes |
| **729** | 999421 | Parabacteroides merdae straub CL09T00C40 | Bacteroidetes |
| **81** | 187327 | Acidaminococcus sp. -- D21 | Firmicutes |
| **182** | 658656 | Clostridiales bacterium -- 4_1_37FAA also known as 6_1_37AA | Firmicutes |
| **84** | 556261 | Clostridiales bacterium -- Deposited as Hespellia sp., Strain 3_1_39B/D5 | Firmicutes |
| **307** | 742731 | Clostridium aldenense -- WAL-18727 | Firmicutes |
| **1038** | 1073368 | Clostridium bolteae -- CC43_001B | Firmicutes |
| **318** | 742732 | Clostridium bolteae -- WAL-14578 | Firmicutes |
| **1040** | 1073356 | Clostridium cadaveris -- CC44_001G | Firmicutes |
| **1041** | 1073357 | Clostridium cadaveris -- CC88A | Firmicutes |
| **315** | 742733 | Clostridium citroniae -- WAL-17108 | Firmicutes |
| **306** | 742735 | Clostridium clostridioforme strain 2_1_49FAA | Firmicutes |
| **317** | 742736 | Clostridium clostridioforme strain WAL-7855 | Firmicutes |
| **746** | 997827 | Clostridium difficile -- 002-P50-2011 | Firmicutes |
| **89** | 525259 | Clostridium difficile -- NAP08 (CDC#2007019) | Firmicutes |
| **88** | 525258 | Clostridium difficile NAP07 (CDC#2007054) | Firmicutes |
| **745** | 1002369 | Clostridium difficile 70-100-2010 | Firmicutes |
| **NA** | 272563 | Clostridioides difficile 630 | Firmicutes |
| **308** | 742737 | Clostridium hathewayi -- WAL-18680 | Firmicutes |
| **173** | 450752 | Clostridium innocuum -- 6_1_30 | Firmicutes |
| **1044** | 1073358 | Clostridium orbiscindens -- CC43_001K | Firmicutes |
| **303** | 742738 | Clostridium orbiscindens strain 1_3_50AFAA | Firmicutes |
| **310** | 742739 | Clostridium perfringens -- WAL-14572 | Firmicutes |
| **36** | 457396 | Clostridium sp. -- 7_2_43FAA | Firmicutes |
| **287** | 908340 | Clostridium sp. -- HGF2 | Firmicutes |
| **309** | 742740 | Clostridium symbiosum strain WAL-14163 | Firmicutes |
| **319** | 742741 | Clostridium symbiosum strain WAL-14673 | Firmicutes |
| **201** | 491074 | Enterococcus faecalis -- TX0104 | Firmicutes |
| **202** | 525278 | Enterococcus faecalis -- TX1322 | Firmicutes |
| **432** | 749491 | Enterococcus faecalis -- TX2137 | Firmicutes |
| **970** | 1134822 | Enterococcus faecium -- ERV165 | Firmicutes |
| **204** | 525279 | Enterococcus faecium -- TX1330 | Firmicutes |
| **178** | 457402 | Eubacterium sp. -- 3_1_31 | Firmicutes |
| **154** | 658082 | Lachnospiraceae sp. -- 2_1_58FAA | Firmicutes |
| **157** | 658085 | Lachnospiraceae sp. -- 5_1_57FAA | Firmicutes |
| **150** | 658083 | Lachnospiraceae sp. -- 6_1_63FAA | Firmicutes |
| **153** | 658087 | Lachnospiraceae sp. -- 7_1_58FAA | Firmicutes |
| **102** | 525341 | Lactobacillus reuteri strain CF48-3A | Firmicutes |
| **106** | 525361 | Lactobacillus rhamnosus strain LMS2-1 | Firmicutes |
| **1056** | 1073375 | Ruminococcus gnavus -- CC55_001C | Firmicutes |
| **1061** | 1073373 | Streptococcus sanguinis -- CC94A | Firmicutes |
| **64** | 457416 | Veillonella sp. -- 3_1_44 | Firmicutes |
| **49** | 450749 | Veillonella sp. -- 6_1_27 | Firmicutes |
| **992** | 1204474 | Fusobacterium nucleatum -- CTI-01 | Fusobacteria |
| **993** | 1204475 | Fusobacterium nucleatum -- CTI-02 | Fusobacteria |
| **995** | 1316586 | Fusobacterium nucleatum -- CTI-05 | Fusobacteria |
| **996** | 1316587 | Fusobacterium nucleatum -- CTI-06 | Fusobacteria |
| **34** | 1639133 | Citrobacter portucalensis -- 30_2 | Proteobacteria |
| **87** | 556266 | Escherichia coli D9 | Proteobacteria |
| **625** | 883119 | Klebsiella oxytoca -- MIT 10-5244 | Proteobacteria |
| **749** | 1203547 | Klebsiella pneumoniae pneumoniae stool from subject with colitis strain WGLW5 | Proteobacteria |
| **748** | 1203546 | Klebsiella pneumoniae pneumoniae stool strain WGLW3 | Proteobacteria |
| **44** | 244366 | Klebsiella sp. -- 1_1_55 | Proteobacteria |
| **223** | 665944 | Klebsiella sp. -- 4_1_44FAA | Proteobacteria |
| **354** | 749535 | Klebsiella sp. -- MS 92-3 | Proteobacteria |
| **214** | 287 | Pseudomonas sp. -- 2_1_26 | Proteobacteria |
| **852** | 1203554 | Sutterella wadsworthensis -- HGA0223 | Proteobacteria |

**Supplementary Table 1 Bacterial strains used in the study.** The first column lists identifiers from the Biodefense and Emerging Infections Research Resources Repository (BEI Resources). The second column lists NCBI taxonomy identifiers. Strain names were retrieved from BEI Resources.

**Supplementary Figure 1. Extracted ion chromatograms (*m/z* 303.1703**) **of caproyl Trp, 3,3-dimethyl butyryl Trp, and *C. difficile* culture.** C6-Trp produced by *C. difficile* is neither caproyl Trp nor 3,3-dimethyl butyryl Trp based on their retention time differences.

**Supplementary Figure 2. Isotope labeling analyses of additional *N*-acyl amino acid features found in *C. difficile* culture shown in Figure 2.** **a.** ^13^C_11_-labeled *N*-acyl tryptophan features found in *C. difficile* culture grown in U-^13^C_11_-tryptophan spiked BHI medium. **b.** ^13^C_9_-labeled *N*-acyl phenylalanine features found in *C. difficile* culture grown in U-^13^C_9_-phenylalanine spiked BHI medium. **c.** ^13^C_9_-labeled *N*-acyl tyrosine features found in *C. difficile* culture grown in U-^13^C_9_-tyrosine spiked BHI medium. Masses corresponding to unlabeled and labeled metabolites are highlighted in red.

**Supplementary Figure 3. MS/MS analyses of unlabeled and isotope labeled *N*-acyl phenylalanine features found in *C. difficile* culture shown in Figure 2.**

**Supplementary Figure 4. MS/MS analyses of unlabeled and isotope labeled *N*-acyl tryptophan features found in *C. difficile* culture shown in Figure 2.**

**Supplementary Figure 5. MS/MS analyses of unlabeled and isotope labeled *N*-acyl tyrosine features found in *C. difficile* culture shown in Figure 2.**

**Supplementary Figure 6. Isotope labeled MS/MS analyses of *N*-phenylacetyl amino acid features found in C. difficile culture shown in Figure 3. a-c.** MS/MS analysis of isotope labeled *m/z* 323 (**a**), *m/z* 300 (**b**), and *m/z* 284 (**c**) features found in *C. difficile* cultures grown in the indicated isotope spiked BHI medium: a1, intact amino acid fragment ions; a2, deaminated amino acid fragment ions; a3, decarboxylated amino acid fragment ions; b, phenylacetyl fragment ions. Superscript indicate isotope labeled fragment ions.

**Supplementary Figure 7. MS/MS and isotope labeling analyses of *N*-phenylpropionyl** **amino acid features found in *C. difficile* culture shown in Figure 3.** **a-c.** Isotope labeling analyses of *m/z* 298 (**d**), *m/z* 337 (**e**), and *m/z* 314 (**f**) features found in *C. difficile* cultures grown in the indicated isotope spiked BHI medium. **d-f.** MS/MS analysis of natural and isotope labeled *m/z* 298 features (**d**). MS/MS analysis of natural and isotope labeled *m/z* 337 features (**e**). MS/MS analysis of natural and isotope labeled *m/z* 314 features (**f**). **g.** Key fragment ions of phenylpropionyl amino acids observed in **d-f**. a1, intact amino acid fragment ions; a2, deaminated amino acid fragment ions; a3, decarboxylated amino acid fragment ions; b, phenylpropionyl fragment ions. Superscript indicate isotope labeled fragment ions.

**Supplementary Figure 8. Comparison of MS/MS spectra of *N*-isocaproyl amino acids and *N*-phenylacetyl amino acids between *C. difficile* HM-746 culture broth to synthetic standards.** **a.** isocaproyl His. **b**. isocaproyl Lys. **c**. isocaproyl Arg. **d**. PAMet. **e.** PALys. **f**. PAArg.

**Supplementary Figure 9. Internal standards showed consistent LC-MS performance across the screen. a-c.** Oligomycin ([M+H]^+^, 791.5311) was spiked into the protein precipitation buffer as internal standard. Mass accuracy (**a**), retention time (**b**), and peak area (**c**) of oligomycin were monitored throughout the analysis. Blank injections were performed every 24 samples, and no significant carryover was observed.
